# Reviews

**Published:** 1864-01

**Authors:** 


### EDITORIAL DEPARTMENT.

### REVIEWS.

*Transactions of the Medical Society of the State of New York*, 1863.

It was late before we received this volume, and notice of it has been necessarily postponed until it is now almost time for the meeting of 1864. The State Medical Society is in reality an organization for the promotion and diffusion of medical knowledge, and the value of its transactions has been yearly increasing until the yearly volume has become one of great practical importance to the profession of the State, to which it is distributed gratuitously through the County Societies and by other media.

We are informed that the delegation from Erie County Medical Society is never full; this we are sorry to learn, for if the County Society is of any value whatever, it consists in forming connection with the Medical Society of the State; if it fails in performing this function it fails of everything useful, and should not be even tolerated, if it has not vitality and vigor enough to do at least one thing valuable. It had two delegates in 1859, and one in 1863. This is the only representation in several years. The

Buffalo Medical College, entitled to one delegate, has been represented only two years in the past eleven, showing about the same inattention to the meetings of the State Society, as that manifested by the Medical Society of the County. We feel a little mortified that so great indifference and neglect should be manifest in Erie County, to the claims and value of the Medical Society of the State, and have called attention to it in this connection with the view to its remedy.*

Returning from our digression, we are to speak of the Transactions of the State Medical Society, and if time and space permitted we should like to give a brief review of each paper presented, of which the volume is composed, but this is uncalled for, since the volume is already in the hands of so many of our readers.

The Annual Address, by Thomas Hune, is the first article, and is "*An inquiry into the degree and kind of influence which the progress of medical science during the present century, has exerted over medical art.*" It is a paper of great interest, expressing the most enlightened views upon the *objects and limits of the art of medicine.* It has been published in pamphlet form and is an article we earnestly recommend to the careful attention of physicians.

*Regimental Surgeons of the State of New York in the War of the Rebellion* 1861–3, by Sylvester D. Willard, M. D., of Albany, is another paper which was presented, and is embraced in the volume of the Transactions. The profession of New York are under lasting obligations to Dr. Willard for his efforts to preserve the names of the physicians who have been engaged in the army from the State. He invites co-operation and proposes to continue the record and make the report full, and correct.—This paper is also in pamphlet form, and is interesting and valuable, constituting a condensed record.

Interesting and remarkable cases with suitable comments by their reporters, introduced mainly in illustration of general principles constitute a considerable portion of the book, which is put up in "State paper" style, but is however creditable for such a document. The Society and the contributors are greatly indebted to the Secretary, Dr. S. D. Willard, for the labor which has been bestowed by him in the preparation of the volume, and though errors and defects are to be noticed, it is yet remarkable under the circumstances of publication, how correct and satisfactory the volume s made to appear.

---

* Since writing the above, we have been assured that it is a mistake, and that the Secretary of the State Society has reported Erie County not represented when delegates were present and their names properly recorded.

*Transactions of the Ohio State Medical Society.*

We are in receipt of the Transactions of the Ohio State Medical Society through the favor of the Secretary, Edward B. Stevens of Cincinnati, to whose efforts the Society are largely indebted for its prosperity, and especially for the value of the yearly volume of Transactions.

The first article consists of a detailed report of the proceedings, the most remarkable part of which are the resolutions, or report adopted concerning the exclusion of calomel and antimony from the supply table of the army. We cannot copy or review, and will pass them over, saying only that they are "*decidedly steep.*"

The second article is the retiring President's Address upon "The cultivation, advancement and elevation of our profession, by J. W. Russell. M. D., of Mt. Vernon. It embodies the usual means recommended for advancing the standard of medical education, and is a plain, practical, common sense address, passing through its different stages, for the most part quite smoothly, but still having some "steep passages" upon exciting objects.

The third article is upon Electricity in Midwifery, by D. S. Gans, M. D., of Cincinnati. Electricity is recommended for "Deficient Labor Pain and Uterine Hæmorrhage," and for "Asphyxia of the New-born." The manner of application is carefully explained.

We then have Report of Committee on New Remedies and on Medical Societies. These are followed by report of cases: "Mollites Ossium, arrest and cure by the use of Phosphate of Lime and Phosphoric Acid," by N. Dalton, M. D., of Logan.

"Remarks upon certain Adipose Tumors," by Alexander McCride, M. D. of Berea, with a list of the members, constitutes the remainder of the volume.

The papers, are many of them, worthy more extended notice, but we have not space to devote to them, and must close by saying that as a whole, the Report of the Transactions is very creditable to the Society.

